# Prevalence and clinical characteristics of malignant lung nodules in tuberculosis endemic area in a single tertiary centre

**DOI:** 10.1186/s12890-022-02125-5

**Published:** 2022-08-29

**Authors:** Norsyuhada Zaharudin, Mas Fazlin Mohamad Jailaini, Nik Nuratiqah Nik Abeed, Boon Hau Ng, Andrea Yu-Lin Ban, Mohd Imree, Rozman Zakaria, Syed Zulkifli Syed Zakaria, Mohamed Faisal Abdul Hamid

**Affiliations:** 1 Respiratory Unit, Department of Medicine, Faculty of Medicine, Universiti Kebangsaan Malaysia Medical Centre, Jalan Yaacob Latif, Bandar Tun Razak, 56000 Kuala Lumpur, Malaysia; 2 Radiology Department, Universiti Kebangsaan Malaysia Medical Centre, Kuala Lumpur, Malaysia; 3 Department of Paediatrics, Universiti Kebangsaan Malaysia Medical Centre, Kuala Lumpur, Malaysia

**Keywords:** Lung nodule, Lung cancer, Tuberculosis

## Abstract

**Background:**

Lung nodule management remains a challenge to clinicians, especially in endemic tuberculosis areas. Different guidelines are available with various recommendations; however, the suitability of these guidelines for the Asian population is still unclear. Our study described the prevalence of malignant lung nodules among nodules measuring 2–30 mm, the demographic and characteristics of lung nodules between benign and malignant groups, and the clinician's clinical practice in managing lung nodules.

**Method:**

Retrospective review of lung nodules from the computed tomography archiving and communication system (PACS) database and clinical data from January 2019 to January 2022. The data was analysed by using chi square, mann whitney test and simple logistic regression.

**Results:**

There were 288 nodules measuring 2–30 mm identified; 49 nodules underwent biopsy. Twenty-seven (55%) biopsied nodules were malignant, (prevalence of 9.4%). Among the malignant lung nodules, 74% were adenocarcinoma (n = 20). The commonest benign nodules were granuloma n = 12 (55%). In nodules > 8 mm, the median age of malignant and benign was 72 ± 12 years and 66 ± 16 years, respectively (*p* = 0.024). There was a significant association of benign nodules (> 8 mm) in subjects with previous or concurrent tuberculosis (*p* = 0.008). Benign nodules are also associated with nodule size ≤ 8 mm, without spiculation (*p* < 0.001) and absence of emphysema (*p* = 0.007). The nodule size and the presence of spiculation are factors to make the clinicians proceed with tissue biopsy. Spiculated nodules and increased nodule size had 11 and 13 times higher chances of undergoing biopsy respectively (*p* < 0.001).) Previous history of tuberculosis had a 0.874 reduced risk of progression to malignant lung nodules (*p* = 0.013). These findings implied that these three factors are important risk factors for malignant lung nodules. There was no mortality association between benign and malignant. Using Brock's probability of malignancy, nodules ≤ 8 mm had a low probability of malignancy.

**Conclusion:**

The prevalence of malignant lung nodules in our centre was comparatively lower than non-Asian countries. Older age, the presence of emphysema, and spiculation are associated with malignancy. Clinical judgment is of utmost importance in managing these patients. Fleishner guideline is still being used as a reference by our clinician.

## Introduction

A lung nodule is a well-defined lesion on imaging measuring < 3 cm, enclosed by lung parenchyma, with no atelectasis, pleural effusion, or lymphadenopathy [1]. Many lung nodules are being discovered with increased computed tomography (CT) utilization, posing a diagnostic challenge, especially in tuberculosis (TB) endemic areas, as this nodule may be benign or malignant.

Lung malignancy may be diagnosed following surveillance of lung nodules from chest imaging or biopsy [2]. It is the most frequent malignancy in men and the leading cause of cancer death in both sexes. It accounts for an estimated 27% of total cancer deaths in the United States in 2015 and 20% in the European Union in 2016 [3]. In Malaysia, there were 11, 256 cases of trachea, bronchus, and lung cancers registered between 2012 and 2016 compared with 10,608 cases in the 2007–2011 report. The majority (68.3%) were males. Lung cancer was the second most common cancer in males and fifth in females. The age-standardized rate (ASR) was highest among Chinese for both sexes, but the trend is increasing among Malays compared to the previous report. The incidence increased with age and peaked at 70 and above. Most lung cancer cases are detected at a very late stage (III & IV), above 90% for both sexes [4]. Hence, it is essential to differentiate benign lung nodules from malignant nodules to identify those eligible for curative surgery.

Management of lung nodules remains a significant challenge. Various guidelines are available with different recommendations, such as Fleischner society and British thoracic society guidelines [5, 6]. The risk of malignancy depends on the patient's risk factor and nodule characteristics. The nodule should be biopsy in high-risk patients [6, 7]. However, the consensus on biopsy is still unclear. Generally, guidelines proposed no follow-up in nodules less than 0.5 cm, radiological surveillance in nodules between 0.5 and 0.8 cm, and biopsy in nodules size more than 0.8 cm depending on the risk [5].

A lung nodule can be a benign infective nodule in the TB endemic area, with different populations having a different probability of malignancy. The American College of Chest Physicians (ACCP) clinical practice guidelines on evaluating lung nodules may not be suitable for Asian countries, given the unique characteristics of the Asian population. This unique characteristic is related to ethnicity, genetics, risk profile, the prevalence of malignant nodules, access to diagnostic services, and cultural understanding of the disease [8].

A clinical practice consensus guideline for the pulmonary nodule was developed in Asia. The decision to biopsy a lung nodule depends on the probability of malignancy and the size and characteristics of the lung nodule. However, the guidelines are not often implemented among clinicians in Asian countries, even though the awareness is high [8]. Multiple tools have been developed to calculate lung malignancy risk, but the strength of association of each risk factor to lung malignancy remains unknown [5, 8, 9].

Our hospital is a tertiary referral center that serves a heterogenous, multiracial population in Kuala Lumpur and TB is common in our catchment area. Being a tertiary centre, CT Scan is done routinely in our centre for various reasons, and many lung nodules are detected incidentally. Hence, we aim to study lung nodules prevalence, characteristics, and outcomes from surveillance computed tomography and biopsy. Our primary objective is to determine the prevalence of malignant lung nodules among nodules measuring 2–30 mm on the CT scan surveillance. Our secondary objective is to describe the characteristics of a malignant lung nodule, determine the risk factors of a malignant lung nodule in our tertiary centre, and assess the clinician's compliance with Fleischner's 2017 guidelines.

## Materials and methods

### Study Design

This study was a single-center, retrospective cohort analysis of CT thorax scans done at Hospital Canselor Tuanku Muhriz (HCTM) between January 2019 and January 2022. Our study was approved by the Research Ethics Committee, Universiti Kebangsaan Malaysia, FF-2021–195. Data were obtained from the radiological picture archiving and communication system (PACS) database and the subject's case notes. We calculated the sample size based on the etiology of a size-based lung nodule in Asia by *SawangSaenghirunvattana *et al*.*" The prevalence (P) of the malignant nodule is 0.25 or 25% [10].

We included subjects with age ≥ 18 years old, lung nodule ranges 2–30 mm from CT thorax, one CT scan available if they underwent biopsy, or at least two CT scan available for assessment of the lung nodules. Subjects with lung mass more than 30 mm and incomplete data were excluded. The interval between two CT scans ranges from one to twelve months, and the shortest follow-up time is 24 months.

### Procedure

CT scan was performed in our center using a CT machine 128-slices Toshiba and 640-slices Acquillon ONE, manufactured in Japan. The slice thickness of CT images is 0.75–1 mm. It used window length − 500 and window width 1500 as setting for observation and the dose of CT scan is 3 mSv. If there are multiple nodules, the largest nodule will be counted.

Subjects who fulfilled this study's inclusion and exclusion criteria were recruited, and demographic and clinical data were collected. These include age, gender, race, occupation, and education level. We also obtained data regarding risk factors for malignancy, such as smoking, tuberculosis, previous history of malignancy, and family history of malignancy.

We divided the nodule into benign and malignant groups based on biopsy results or size stability on CT scans. For simplicity, a subanalysis was done based on lung nodule sizes ≤ 8 mm and > 8 mm. We chose 8 mm because most guidelines suggest one or a combination of three options in assessing nodules > 8 mm: 3-month follow-up, Positron emission tomography (PET)-CT, or tissue biopsy, with no particular guidance on the selection of choice [5, 7].

CT characteristics of nodules such as number, size, density, location, and spiculation were recorded. We took the most significant size in subjects with multiple lung nodules. The findings of the CT thorax was based on the radiologist's report and the consultant radiologist confirmed all the findings. Data on subjects who had undergone Positron emission tomography (PET)-CT scan were also recorded.

The biopsy mode was recorded in the benign and malignant groups when available: whether early biopsy or after the surveillance scan. We defined early biopsy as when the biopsy was done within three months or after the first CT scan. Complications of the biopsy were documented in both groups. We also evaluated the clinician's adherence based on their management decision.

Our study used the Brocks model to evaluate the probability of malignancy in each subject [11]. Subjects were categorized into low, intermediate, and high risk. Low risk is when the calculated risk is less than 5%, while the intermediate risk is 5–65%, and high risk > 65% [7].

## Statistical analysis

Data were analysed using SPSS software version 28.0. All categorical variables were presented as frequencies and percentages. Data without normal distribution were expressed as the median and interquartile range (IQR). Mann Whitney test was used for comparison of difference in the dependent variable for two independent groups, while chi-square and Fishers' exact test were used to compare two categorical data variable. The logistic regression was used to predicts a dependent data variable by examining the relationship between one or more existing independent variables. The *p* < 0.05 is considered significant.

## Results

**Fig. 1:**
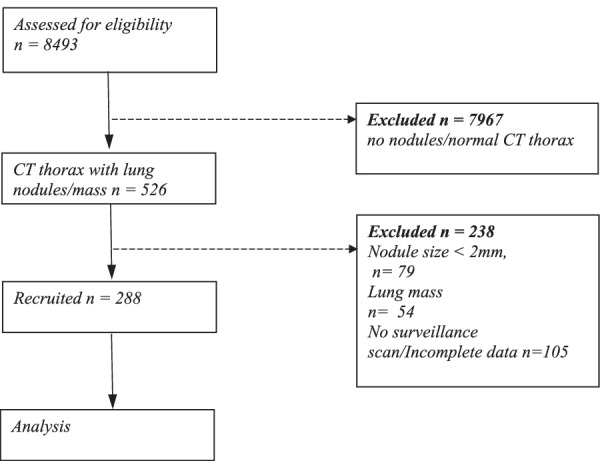
The flow chart of the study

There was a total of 8493 CT Scan thorax done between Jan 2019 and January 2022. After excluding 8205 patients for various reasons, 288 patients with lung nodules were recruited. Figure 1 shows the study flow chart.

Table 1 described the demographic and characteristics of benign and malignant nodules. The median age of the study subjects was 66 ± 15 years old, and 143 (49.7%) were male. The majority were Malays 143 (49.7%), followed by Chinese 113 (39.2%) and Indians 29 (10.1%). Most subjects had secondary education, 137 (47.6%), and worked as sales workers and services 62 (21.5%). 206 (71.5%) of the total population were non-smokers. There were 58 (20.1%) of the subjects who had underlying or previous pulmonary tuberculosis, 84 (29.2%) with extrathoracic malignancy, and 49 (17%) with chronic lung disease.

**Table 1:** The demographic and characteristics of benign and malignant nodules according to the size

Total population n (%) n = 288 (100%)	Nodule ≤ 8 mm, n (%) n = 188 (65%)		*p*-value	Nodule > 8 mm, n (%) n = 100 (35%)		*p*-value
	Benign (size stability on CT Scan) n = 186 (64%)	Malignant (biopsy proven) n = 2 (1%)		Benign (size stability on CT Scan) n = 53 (18%) Biopsy n = 22 (8%)	Malignant (biopsy proven) n = 25 (9%)	
Age, median (IQR)	66 (16)	70 (–)	0.460^b^	66 (16)	72 (12)	0.024^b^
*Gender*						
Male	87 (98.9)	1 (1.1)	0.718^a^	43 (78.2)	12 (21.8)	0.417
Female	99 (99.0)	1 (1.0)		32 (71.1)	13 (28.9)	
*Ethnicity*						
Malay	93 (100.0)	0	0.312	36 (72.0)	14 (28.0)	0.487
Chinese	66 (97.1)	2 (2.9)		36 (80.0)	9 (20.0)	
Indian	24 (100.0)	0		3 (60.0)	2 (40.0)	
Others	3 (100.0)	0		0	0	
*Marital*						
Single	13 (92.9)	1 (7.1)	0.069	8 (100.0)	0	0.186
Married	165 (99.4)	1 (0.6)		64 (73.6)	23 (26.4)	
Divorced/Widowed	8 (100.0)	0		3 (60.0)	2 (40.0)	
*Education*						
Primary education	48 (100.0)	0	0.740	24 (68.6)	11 (31.4)	0.558
Secondary education	93 (97.9)	2 (2.1)		31 (73.8)	11 (26.2)	
Certificate	9 (100.0)	0		2 (100.0)	0	
Tertiary education	34 (100.0)	0		16 (84.2)	3 (15.8)	
No education	2 (100.0)	0		2 (100.0)	0	
*Smoking*						
Active smoker	14 (100.0)	0	0.606	10 (90.9)	1 (9.1)	0.613
Non-smoker	136 (99.3)	1 (0.7)		50 (72.5)	19 (27.5)	
Ex-smoker						
Stop < 15 years	7 (100.0)	0		4 (80.0)	1 (20.0)	
Stop > 15 years	29 (96.7)	1 (3.3)		11 (73.3)	4 (26.7)	
Heavy alcohol*						
Yes	2 (100.0)	0	0.979^a^	0	0	–
No	184 (98.9)	2 (1.1)		75 (75.0)	25 (25.0)	
*Occupations*						
Professionals	19 (100.0)	0	0.656	7 (87.5)	1 (12.5)	0.238
Technicians	7 (100.0)	0		3 (75.0)	1 (25.0)	
Clerks	18 (100.0)	0		7 (50.0)	7 (50.0)	
Service and sales workers	41 (95.3)	2 (4.7)		16 (84.2)	3 (15.8)	
Skilled agricultural, Forestry/ fishery workers	3 (100.0)	0		1 (100.0)	0	
Craft and related trade workers	20 (100.0)	0		9 (69.2)	4 (30.8)	
Plant and machine operators and assemblers	5 (100.0)	0		3 (60.0)	2 (40.0)	
Elementary occupation	16 (100.0)	0		11 (100.0)	0	
Housewife	55 (100.0)	0		17 (70.8)	7 (29.2)	
Student	2 (100.0)	0		1 (100.0)	0	
*Family history of lung malignancy,*						
Yes	2 (100.0)	0	0.979^a^	2 (100.0)	0	0.561^a^
No	184 (98.9)	2 (1.1)		73 (74.5)	25 (25.5)	
*Underlying/Previous history of tuberculosis*						
Yes	29 (100)	0	0.715^a^	27 (93.1)	2 (6.9)	0.008
No	157 (98.7)	2 (1.3)		48 (67.6)	23 (32.4)	
*Underlying/Previous history of extrathoracic malignancy*						
Yes	66 (100.0)	0	0.420^a^	15 (83.3)	3 (16.7)	0.367
No	120 (98.4)	2 (1.6)		60 (73.2)	22 (26.8)	
*Underlying Chronic lung disease*^#^						
Yes	34 (100.0)	0	0.670^a^	13 (86.7)	2 (13.3)	0.214^a^
No	152 (98.7)	2 (1.3)		62 (72.9)	23 (27.1)	
*Outcome*						
Alive	171 (98.8)	2 (1.2)	0.846^a^	67 (76.1)	21 (23.9)	0.347^a^
Death	15 (100)	0		8 (66.7)	4 (33.3)	
*PET*						
Yes	3 (100.0)	0	0.968^a^	7 (50.0)	7 (50.0)	0.027^a^
No	183 (98.9)	2 (1.1)		68 (79.1)	18 (20.9)	
*Location*						
Upper lobe	82 (100.0)	0	0.240	43 (79.6)	11 (20.4)	0.449
Middle lobe	26 (100.0)	0		5 (62.5)	3 (37.5)	
Lower lobe	59 (96.7)	2 (3.3)		27 (71.1)	11 (28.9)	
All lobes	19 (100.0)	0		0	0	
*Multiplicity*						
Single nodule	36 (100.0)	0	0.653^a^	18 (78.3)	5 (21.7)	0.681
Multiple nodules	150 (98.7)	2 (1.3)		57 (74.0)	20 (26.0)	
*Density*						
Solid	174 (98.9)	2 (1.1)	0.933	70 (73.7)	25 (26.3)	0.625
Subsolid	12 (100.0)	0		5 (100.0)	0	
*Presence of calcification*						
Yes	41 (97.6)	1 (2.4)	0.398^a^	12 (92.3)	1 (7.7)	0.110^a^
No	145 (99.3)	1 (0.7)		63 (72.4)	24 (27.6)	
*Presence of spiculation*						
Yes	2 (66.7)	1 (33.3)	< 0.001	18 (60.0)	12 (40.0)	0.023
No	184 (99.5)	1 (0.50)		57 (81.4)	13 (18.6)	
*Emphysema*						
Yes	10 (90.9)	1 (9.1)	0.007	4 (80.0)	1 (20.0)	0.633^a^
No	176 (99.4)	1 (0.6)		71 (74.4)	24 (25.3)	
*Probability of malignancy ¶*						
Low (< 5%)	184 (98.9)	2 (1.1)	0.979^a^	8 (100.0)	0	0.234
Intermediate(5–65%)	2 (100.0)	0		64 (72.7)	24 (27.3)	
High (> 65%)	–	–		3 (75.0)	1 (25.0)	

^a^Fishers exact test ^b^Mann-Whitney test. Other analyses using Pearson chi-square

^*^Heavy alcohol: 15 drinks or more per week in males and 8 drinks or more per week in females

^#^ Chronic lung disease – Interstitial lung disease, Lung fibrosis, Bronchiectasis

^¶^ Based on Brock's probability of malignancy

In our study, the prevalence of malignant lung nodules among nodule sizes 2–30 mm was 9.4% (27 out of 288 patients).

Our study compared demographic details and nodules characteristics between benign and malignant nodules. We classified lung nodules according to their size; out of 188 (65%) nodules ≤ 8 mm, 186 (64%) nodules sizes remained stable with CT surveillance, and 2 (1.0%) nodules were biopsy-proven malignant. There were 100 (35%) nodules > 8 mm; 75 (26%) nodules were benign based on CT surveillance stability and biopsy, with 25 (9%) nodules being malignant.

There was no demographic difference between benign and malignant lung nodules in the nodules ≤ 8 mm. However, in nodules > 8 mm, the mean age of malignant and benign was 72 ± 12 years and 66 ± 16 years (*p* = 0.024), respectively There was a significant association of benign nodules (> 8 mm) in subjects with previous or concurrent tuberculosis (*p* = 0.008). Benign nodules are associated with nodule size ≤ 8 mm and without spiculation (*p* < 0.001) or absence of emphysema (*p* = 0.007) (Table 1).

There was no mortality association between benign and malignant nodule. Using Brock's probability of malignancy, nodules ≤ 8 mm had a low probability of malignancy.

Table 2 illustrates that nodule size and the presence of spiculation are factors to make the clinicians proceed with tissue biopsy. In addition, increased nodule size had 13 higher chances of biopsy (*p* < 0.001). Presence of spiculation had 11 times increased chances to undergo a biopsy (*p* < 0.001). The previous history of tuberculosis had a 0.874 reduced risk of proceeding to tissue biopsy (*p* = 0.013). These findings implied that these three factors are important risk factors for malignant lung nodules.

**Table 2:** Risk of malignant nodule according to the decision of lung nodules biopsy

Total population n (%) *n* = 288 (100%)	Simple logistic regression		
	b	Crude OR (95% CI)	*p*-value
Age, median (IQR)	0.006	1.006 (0.967,1.046)	0.768
Nodule size	2.582	13 (6.399,27.344)	< 0.001
Underlying/previous history of tuberculosis	− 1.539	0.874 (0.131,5.811)	0.013
Presence of spiculation	2.427	11.328 (3.703,34.654)	< 0.001
Emphysema	− 0.135	0.874 (0.131,5.811)	0.889

Table 3 illustrates the biopsy group's characteristic difference between a malignant and benign nodule. A total of 49 lungs nodules underwent biopsy. Out of 49 biopsy nodules, 27 (55%) were malignant. The univariate analysis of risk factors and characteristics of malignant lung nodules showed no significant difference between these two groups. The majority of biopsies methods were CT-guided biopsies (n = 46). Eight subjects developed complications, and the commonest was pneumothorax (n = 7). There was no difference in complications occurrence post-biopsy of a benign and malignant nodule.

**Table 3:** The characteristics of biopsied benign and malignant nodules

Characteristics	Benign, *n* (%) *n* = 22 (45%)	Malignant, *n* (%) *n* = 27 (55%)	*p*-value
Age, median (IQR)	68.5 (17.5)	72 (11)	0.144^b^
*Sex, n (%)*			
Male	15 (53.6)	13 (46.4)	0.159
Female	7 (33.3)	14 (66.7)	
*Family history of Lung cancer, n (%)*			
Yes	1 (100.0)	0	0.449^a^
No	21 (43.8)	27 (56.3)	
*Smoking, n (%)*			
Active smoker	6 (85.7)	1 (14.3)	0.087
Non-smoker	11 (35.5)	20 (64.5)	
*Ex-smoker*			
Stop < 15 years	2 (66.7)	1 (33.3)	
Stop > 15 years	3 (37.5)	5 (62.5)	
Nodule size (cm), median (IQR)	1.7 (1.02)	2.0 (1.4)	0.146^b^
*Location, n (%)*			
Upper lobe	15 (57.7)	11 (42.3)	0.155
Middle lobe	1 (25.0)	3 (75.0)	
Lower lobe	6 (31.6)	13 (68.4)	
*Multiplicity, n (%)*			
Single nodule	6 (54.5)	5 (45.5)	0.348^a^
Multiple nodules	16 (42.1)	22 (57.9)	
*Density, n (%)*			
Solid	22 (44.9)	27 (55.1)	–
Subsolid	0	0	
*Presence of spiculation, n (%)*			
Yes	10 (43.5)	13 (56.5)	0.851
No	12 (46.2)	14 (53.8)	
*Emphysema, n (%)*			
Yes	1 (33.3)	2 (66.7)	0.678^a^
No	21 (45.7)	25 (54.3)	
*Biopsy method, n (%)*			
CT Guided biopsy	22 (47.8)	24 (52.2)	0.242^a^
Others*	0	3 (100.0)	
*Complications, n (%)*			
Yes			
Pneumothorax	1 (14.3)	6 (85.7)	0.127
Subcutaneous emphysema	1 (100.0)	0	
No	20 (48.8)	21 (51.2)	

^a^Fishers exact test ^b^Mann-Whitney test. Other analyses using Pearson chi-square test

*Others, i.e., Transbronchial needle biopsy

Table 4 illustrates the distribution of histopathology results. In our study, the commonest malignant nodule was adenocarcinoma 20/27 nodules (74%) and commonest benign nodule was granuloma 12/22 nodules (55%).

**Table 4:** The distribution of Histopathology results of biopsied nodule

Malignant nodules. *n* = 27 (55%)	*n* (100%)^a^
Primary	
Small cell carcinoma	1 (2%)
Squamous cell carcinoma	3 (6%)
Adenocarcinoma	20 (40%)
Others	
Neuroendocrine (carcinoid)	1 (2%)
Metastatic—adenocarcinoma^b^	2 (5%)
Benign nodules, *n* = 22 (45%)	
Chondroid hamartoma	1 (2%)
Benign tissue	2 (5%)
Granuloma	12 (24%)
Necrotic tissue/Necrotic hyalinized stroma	6 (12%)
Fibrocollagenous + lymphoplasmacytic infiltration	1 (2%)

^a^The number (100%) represents total nodules (benign and malignant)

^b^Pancreatic and thyroid cancer

Table 5 compares nodules size, probability of malignancy, PET scans availability, and lung nodules outcome between early and late biopsy. 76.6% of the nodules sized more than 8 mm underwent early biopsy n = 36. In addition, the majority of biopsied nodules had an intermediate probability of malignancy. Our study showed a significant difference in PET scan availability between these two groups (*p* < 0.001). More patients with late biopsy underwent PET scans than the early biopsy group. Otherwise, there is no difference in lung nodules outcome between these two groups.

**Table 5:** The comparison between early and late biopsy

Total numbers of biopsy (n = 49)	Early biopsy* *n* (%) *n* = 38 (77.6)	Late biopsy** *n* (%) *n* = 11 (22.4)	*p*-value
*Nodule size*			
2-8 mm	2 (100.0)	0	0.598^a^
8.1–30 mm	36 (76.6)	11 (23.4)	
*Probability of malignancy*			
< 5% (Low)	2 (100.0)	0	0.447
5–65% (Intermediate)	22 (75.0)	11 (25.0)	
> 65% (High)	3 (100.0)	0	
*PET scan available*			
Yes	2 (20.0)	8 (80.0)	< 0.001
No	36 (92.3)	3 (7.7)	
*Nodule outcome group*			
Benign	16 (72.7)	6 (27.3)	0.348^a^
Malignant	22 (81.5)	5 (18.5)	

^a^Fishers exact test. Other analyses using Pearson chi-square test

*Early biopsy—Biopsy after first CT scan or within three months of CT scan

**Late biopsy—Biopsy after CT scan surveillance

Table 6 describes the clinician's compliance with Fleishner society pulmonary nodule surveillance guidelines. In our study, most nodules sized ≤ 8 mm, and low probability of malignancy underwent surveillance scan. In addition, the majority of nodules > 8 mm had an intermediate probability of malignancy n = 47 (47%) underwent tissue biopsy. This study shows that clinicians are still complying with Fleishner guideline.

**Table 6:** Clinician's compliance to Fleischner society pulmonary nodule guideline

	Nodule ≤ 8 mm, *n* (%) *n* = 188 (65%)	Nodule > 8 mm, *n* (%) *n* = 100 (35%)
*Probability of malignancy*		
< 5% (Low)	186 (98.9)	8 (8.0)
5–65% (Intermediate)	2 (1.1)	88 (88.0)
> 65% (High)	0	4 (4.0)
*Biopsy*		
Yes	2 (1.1)	47 (47.0)
No	186 (98.9)	53 (53.0)
*CT surveillance, according to Fleischner pulmonary nodule guideline*		
Yes	44 (24.0)	75 (75.0)
No*	144 (76.0)	25 (25.0)

*Fleishners guideline is not applicable (Nodules size < 0.5 cm, Age < 35 years old, extrathoracic malignancy)

## Discussions

Solitary lung nodule represents a radiological entity with unknown prevalence in the general population. There are multiple guidelines and tools for the assessment of lung nodules. However, different guidelines have different recommendations. Some guidelines excluded particular conditions. For example, the Fleischner society guideline excluded patients younger than 35, immunocompromised, and with underlying malignancy [7]. For assessment of lung nodules, PET/CT Scan is done routinely in some centres. However, PET-scan imaging needs to be correlated with the clinical situation. Patients with PET-negative nodules may still have malignancy and follow-up beyond two years is recommended [12]. On the other hand, it is difficult to differentiate between infective and malignant nodules from PET/CT as both will have high uptake.[6, 7, 13].

The existence of local guidelines on the management of lung nodules differs widely throughout different countries in Asia. The decision to manage lung nodules depends on the clinician's clinical judgment. The clinician must decide between observation with surveillance imaging or biopsy based on the clinical probability. There are several classification models to classify the malignancy probability of lung nodules. Our study used the Brock model, also known as the PanCan model. The model developed from participants enrolled in the Pan-Canadian Early Detection of Lung Cancer Study has been validated in lung cancer screening and clinical populations and is recommended by the British Thoracic Society guidelines for pulmonary nodules.[11] They divide the probability of malignancy into three categories which are Low (less than 5%), Intermediate probability (5% to 65%), and High probability (greater than 65%) [14, 15]. Our study shows a greater number of nodules that less than 8 mm had a very low probability of malignancy. In comparison, nodules more than 8 mm had an intermediate probability of malignancy. Hence the majority of malignant nodules were in this group. This study reflects that the probability of malignancy is a reliable tool to assist clinicians in managing lung nodules.

There are multiple literature reviews analyzing lung nodule's etiology, correlating it with clinical and radiological aspects. Turpin. S. et al. reviewed 119 cases of lung nodules. In half of the nodules, 54% of the diagnosis made was a malignancy. However, the average malignant nodules size is 2.8 + / − 10.9 cm [16]. In another study published by Motaş. N. et al., among 150 resected solitary pulmonary nodules, 48.66% are malignant, and 52.66% of the nodules have the maximum accepted dimension of 3 cm [17].

Malaysia does not have a lung cancer screening program. The majority of lung nodules were detected incidentally. Our study reviewed lung nodules size 2–30 mm. We took all the lung nodules with at least two CT scans available for assessment. Guidelines recommended no surveillance scan in lung nodules less than 5 mm. [5] Most nodules less than 5 mm had CT scans for other reasons, including assessing lung fibrosis, lung infection, or pulmonary embolism with incidental tiny lung nodules. The majority of these nodules resolved, or the size remained stable. In our study, the prevalence of malignant lung nodules among nodule sizes 2–30 mm was 9.4%. This prevalence is slightly lower than malignant lung nodules in other studies and could be due to the broader range of observed nodule size.

There was no significant difference comparing the demographic data between benign and malignant in both groups apart from age. An increase in age and smoking is associated with an increased risk of lung malignancy [4, 18]. However, our study showed that many non-smokers develop cancer. A total of 71.5% (n = 206) of the study population were non-smokers, and 64.5% of lung cancer patients were non-smokers (n = 20). (Table 3) This data is consistent with a study by Phua et al. looking at pulmonary nodules in the Asian population [19]. Our study has similar findings to other studies in which older age is associated more with malignant lung nodules [11].

Marital and education are also important in predicting disease survival. One study by Goodwin, et al. showed that marital status affected the treatment and survival of patients with cancer. They conclude that unmarried persons were more likely to be diagnosed at a regional or distant stage and untreated for cancer [20]. However, no review of the association between marital status and the risk of malignant lung nodules. There are research studies on the relationship between occupation and lung cancer. Some professions had exposure to carcinogenic substances. For example, machine operators, service workers, and elementary occupations [21]. However, there was no significant difference between occupation and outcome of lung nodules in our study. It might be due to no proper assessment of the strength of carcinogenic exposure in each occupation.

Another challenge in the assessment of lung nodules is differentiating malignancy and tuberculosis. Tuberculosis is historically a well-known disease. It has a similar presentation and radiological features. This similarity often leads the Physician to suspect tuberculosis in a patient with lung nodules, causing misdiagnosis. The incidence of tuberculosis in western countries is lower compared to Asian countries [19]. Malaysia is one of the TB endemic areas.

In our study population, 20.1% had previous or concurrent tuberculosis. Our study showed a significant difference between tuberculosis and the outcome of lung nodules; 77.8% of patients with tuberculosis had benign nodules, and 22.2% had malignant nodules (*p* = 0.034)(Table 1). This result is probably due to the clinicians being familiar with tuberculosis and differentiating between this disease. Furthermore, this study highlights no difference in nodule characteristics except the presence of spiculation and emphysema, which were associated with the malignant nodule.

In a patient with operated and stable extrathoracic malignancy, surveillance imaging sometimes showed new lung nodules with no local recurrence. Assessment of lung nodules in existent extrathoracic malignancy is complex. It is difficult to differentiate between metastatic nodules and benign nodules. Several classification models classify the malignancy probability of lung nodules in the literature, e.g., Mayo clinic, veterans affairs, and Brock University [22]. The Mayo clinic model excludes primary lung cancer and extrathoracic malignancy within five years of nodules presentation. Hence this model cannot be use it on a patient who recently diagnosed with cancer.

A study reviews the outcome of lung nodules biopsy in a patient with extrathoracic malignancy. They found that multiple lung nodules of more than 0.5 cm and cavitation were two characteristics associated with high chances of metastatic disease [23].Our study showed that 83.3% of nodules > 8 mm in patients with underlying extrathoracic malignancy had remained stable (n = 15), and only 16.7% of patients were diagnosed with a second primary lung tumor (n = 3) (Table1). There is no association between extrathoracic malignancy and the outcome of a lung nodule. The difference in the result is probably due to most of our patients having stable extrathoracic cancer. Hence, the clinicians decided to observe the nodules, and the nodule size remained stable in the surveillance scan. However, most guidelines exclude extrathoracic malignancy in their tool assessment; hence, the clinician's judgment is crucial.

Patients with chronic lung disease usually have surveillance CT scans for disease assessment. Surveillance scans generally detect the presence of lung nodules. Our study showed that 17% of patients had chronic lung disease n = 49 (Table 1). There is no association of lung disease with the outcome of lung nodules.

Following ACCP guidelines, it recommends a PET scan following the pre-test probability calculation. Our center has done fewer PET scans compared to western countries. Seven nodules in non-malignant groups and 7 in malignant nodules size more than 0.8 cm had a PET scan. This number illustrates that our Physician relies less on a PET scan for malignant nodule assessment. It could be due to the increased chance of false-positive PET scans in tuberculosis endemic areas, rendering it less relevant in the Asian population [19].

Our study showed that most tissue biopsies were done via CT-guided image biopsy n = 22 (47.8%) in benign nodules and n = 24 (52.2%) in malignant nodules. Most of the complications happened in subjects with CT-guided biopsy. This result could be due to the less feasibility of other biopsy methods. Newer technology arises, for example, radial endobronchial ultrasound (r-EBUS). However, it is not widely available. Malaysia Health technology assessment (MaHTAS) concludes that bronchoscopic techniques have a safety profile, especially the risk of procedure-related pneumothorax and hemorrhage [24].

One of the objectives of this study is to assess clinicians' compliance with available guidelines. A study was carried out to determine radiologists' familiarity with the Fleischner guidelines and their decision for nodules management. They conduct the study via electronic survey and questionnaire. They found out there was high awareness and adoption of the Fleischner guidelines among survey respondents, but radiologists showed varying agreement with these recommendations [25]. Our study does not explicitly assess the compliance of radiologists and clinicians toward available guidelines. We did not do a questionnaire or interview. The clinicians' adherence to the guideline was evaluated based on their management decision. Our study found that 24% (n = 44) of nodules ≤ 8 mm while 75% (n = 75) of nodules size > 8 mm had CT surveillance according to Fleischner guidelines. The lower percentage in the ≤ 8 mm group is due to many nodules having a size less than 5 mm.

Our study compares the size, probability of malignancy, and the outcome of the lung nodules between early and late biopsy. It shows no significant difference in all these parameters. However, most biopsy nodules have a size of more than 0.8 cm and a low/moderate probability of malignancy. However, PET/CT is not widely used. This management aligns with some of the recommendations and indicates that clinicians comply with them.

Our study possessed some limitations. Our study found no significant difference in nodule characteristics between benign and malignant nodules. There may be a type 1 error as our sample size for biopsy nodules was small. Another limitation is that our study was conducted in a single center and may not represent the actual population. Furthermore, CT scans were not part of the lung cancer screening program, causing us to underestimate the number of nodule detection.

In conclusion, our center's prevalence of malignant lung nodules was comparatively lower than non-Asian countries. Increasing age, emphysema, and spiculation are associated with malignancy. CT surveillance might be an option compared to biopsy in nodule size > 8 mm in a patient with the previous TB. Hence clinical judgment is of utmost importance in managing these patients. Fleishner guidelines are still being used as a reference by our clinician.

## Data Availability

The datasets used and/or analysed during the current study available from the corresponding author on reasonable request.

## Abbreviations

TB: Tuberculosis
CT: Computed tomography
PET: Positron emission tomography

## References

[1] Larici AR (2017). Lung nodules: size still matters. Eur Respir Rev.

[2] Loverdos K (2019). Lung nodules: a comprehensive review on current approach and management. Ann Thorac Med.

[3] Malhotra J (2016). Risk factors for lung cancer worldwide. Eur Respir J.

[4] Azizah AM, Hashimah B, Nirmal K et al. Malaysian national cancer registry (MNCR) Report 2012–2016. Available at http://nci.moh.gov.my

[5] Callister MEJ (2015). British thoracic society guidelines for the investigation and management of pulmonary nodules: accredited by NICE. Thorax.

[6] MacMahon H (2005). Guidelines for management of small pulmonary nodules detected on CT scans: a statement from the Fleischner society. Radiology.

[7] MacMahon H (2017). Guidelines for management of incidental pulmonary nodules detected on CT images: from the Fleischner society 2017. Radiology.

[8] Bai C (2016). Evaluation of pulmonary nodules: clinical practice consensus guidelines for Asia. Chest.

[9] Chang CF, Gould MK (2016). The importance of being adaptable: developing guidelines for lung nodule evaluation in Asia. Chest.

[10] Saenghirunvattana S, Kurimoto N, Suwanakijboriharn C, Napairee C, Pitiguagool V, Saenghirunvattana C, Gonzales MC, Sutthisri K, Siangproh C (2016). Etiology of size based pulmonary nodules in Asia. Bangk Med J.

[11] McWilliams A (2013). Probability of cancer in pulmonary nodules detected on first screening CT. N Engl J Med.

[12] Chen J (2020). Outcome of PET-negative solid pulmonary nodules: a retrospective study. Acad Radiol.

[13] Horeweg N (2014). Lung cancer probability in patients with CT-detected pulmonary nodules: a prespecified analysis of data from the NELSON trial of low-dose CT screening. Lancet Oncol.

[14] Swensen SJ (1999). Solitary pulmonary nodules: clinical prediction model versus physicians. Mayo Clin Proc.

[15] Balekian AA (2013). Accuracy of clinicians and models for estimating the probability that a pulmonary nodule is malignant. Ann Am Thorac Soc.

[16] Turpin S (1998). The solitary pulmonary nodule. A retrospective study of 119 cases. Acta Med Port.

[17] Motaş N (2010). Solitary pulmonary nodule–150 resected cases. Chirurgia (Bucur).

[18] Furrukh M (2013). Tobacco smoking and lung cancer: perception-changing facts. Sultan Qaboos Univ Med J.

[19] Phua CK (2016). Evaluation of pulmonary nodules in Asian population. J Thorac Dis.

[20] Goodwin JS (1987). The effect of marital status on stage, treatment, and survival of cancer patients. JAMA.

[21] Consonni D (2010). Lung cancer and occupation in a population-based case-control study. Am J Epidemiol.

[22] Cui X (2019). Comparison of Veterans Affairs, Mayo, Brock classification models and radiologist diagnosis for classifying the malignancy of pulmonary nodules in Chinese clinical population. Transl Lung Cancer Res.

[23] Caparica R (2016). Pulmonary nodules in patients with nonpulmonary cancer: not always metastases. J Glob Oncol.

[24] Fuzi, Msabm and Dezb Romli, *Diagnostic approaches to solitary pulmonary nodule (SPN)*. November 2020: Malaysia

[25] Eisenberg RL, Bankier AA, Boiselle PM (2010). Compliance with Fleischner society guidelines for management of small lung nodules: a survey of 834 radiologists. Radiology.

